# Clinical and imaging findings of neurosyphilis

**DOI:** 10.3389/fneur.2025.1655101

**Published:** 2025-09-19

**Authors:** Peng He, Wen-hui Ma, Jie Jiang, Wen-sheng Wang, Cong Huang, Hong Bai

**Affiliations:** ^1^Department of Imaging, Guangdong Sanjiu Brain Hospital, Guangzhou, Guangdong, China; ^2^Department of Radiology, No. 926 Hospital, Joint Logistics Support Force of PLA, Kaiyuan, Yunnan, China; ^3^Department of Medical Imaging, The First Affiliated Hospital of Kunming Medical University, Kunming, Yunnan, China; ^4^Department of Neurology, No. 926 Hospital, Joint Logistics Support Force of PLA, Kaiyuan, Yunnan, China

**Keywords:** neurosyphilis, universal imitator, syphilis gumma, magnetic resonance imaging, parenchymal type, meningovascular type

## Abstract

**Background:**

This study aimed to systematically analyze the clinical and MRI characteristics of four types of neurosyphilis to improve diagnostic accuracy and facilitate early treatment. By deepening the understanding of clinical presentations and MRI findings, this study seeks to enhance differential diagnosis capabilities.

**Methods:**

This was a retrospective study analyzing clinical and MRI data from 23 patients diagnosed with neurosyphilis between January 2016 and May 2024. MRI examinations were performed using 3-Tesla scanners (Siemens, Germany; General Electric, United States) with an 8-channel head coil. The imaging protocol included T1-weighted imaging (T1WI), T2-weighted imaging (T2WI), fluid-attenuated inversion recovery (FLAIR), diffusion-weighted imaging (DWI), and contrast-enhanced T1-weighted imaging (T1WI-CE) using gadolinium-based contrast agents.

**Results:**

Of the 23 cases of neurosyphilis, including 19 males and 4 females, the mean age was 49.2 ± 11.4 years (range: 27–67). Sixteen cases of parenchymal type (69.6%), mainly manifested as progressive cognitive impairment with psycho-behavioral abnormalities. MRI mainly showed bilateral temporal lobe and hippocampal atrophy with signal abnormalities, with or without abnormal signals in other brain parenchyma, and the enhancement patterns were diverse, which may be unenhanced, patchy, or strip enhancement. Three cases of meningovascular type (13.0%), presented with ischemic stroke with short duration and acute onset. MRI mainly showed multiple acute cerebral infarcts with extensive but scattered intracranial lesions. Three cases of syphilis gumma type (13.0%), had a long course of disease and mainly presented with headache. MRI mainly showed multiple lesions in the internal cerebral convexity with significant surrounding edema bands and significant enhancement of the enhanced lesions with adjacent meningeal enhancement. Mixed type in 1 case (4.4%), presented with headache. MRI findings were complex.

**Conclusion:**

The clinical and MRI manifestations of neurosyphilis are diverse, with significant variations among subtypes. Quantitative imaging biomarkers, including lesion volume and SIR, demonstrated diagnostic utility, particularly in distinguishing parenchymal and meningovascular types. Integrating these biomarkers with clinical evaluation may improve diagnostic precision and facilitate targeted interventions.

## Introduction

Neurosyphilis is a chronic neurological disorder caused by the invasion of *Treponema pallidum* into the nervous system, typically resulting from insufficient treatment of early syphilis. It represents a serious complication of advanced (stage III) syphilis, with an incidence ranging from 0.47 to 2.1 cases per 100,000 people (1, 2). Recent research indicates neurosyphilis can manifest at any time post-infection (3), with a prevalence of 1.8% among early syphilis patients in the United States (4). Despite effective control with penicillin, factors such as increased population mobility, evolving sexual behaviors -particularly among men who have sex with men (MSM)- and HIV co-infection have contributed to its resurgence over the past two decades (5, 6). Robust epidemiological evidence indicates that MSM are disproportionately affected by primary and secondary syphilis, with transmission often facilitated by high-risk sexual networks, concurrent sexually transmitted infections (notably HIV), and behavioral patterns that enhance transmission efficiency. This provides strong scientific support for the role of this population in the current resurgence of syphilis (5, 7–9).

Due to its varied clinical symptoms and diverse MRI presentations, neurosyphilis is often termed a “Great Imitator.” Clinical manifestations range from asymptomatic to ischemic stroke, cognitive impairment, and psychiatric syndromes. MRI findings may include encephalitis, cerebral infarction, inflammatory granulomas, or tumor-like changes, leading to frequent initial misdiagnosis and delayed treatment (10).

Timely recognition of MRI findings is essential for accurate diagnosis, as cognitive impairment caused by neurosyphilis may be reversible. However, comprehensive MRI classification and reporting remain scarce, with most existing studies being limited to individual case reports. This study aims to categorize clinical symptoms and MRI findings into four types, providing analysis of each type’s symptoms, MRI characteristics, and relevant differentials to enhance clinical awareness and optimize treatment selection.

## Materials and methods

### Patient

This retrospective study was approved by the Ethics Committee of No. 926 Hospital, Joint Logistics Support Force of PLA (2024–002), and it conformed to the ethical standards for medical research in The Declaration of Helsinki. The requirement for informed consent was exempted due to the retrospective nature of this study.

In total, 23 patients with neurosyphilis from January 2016 to May 2024 were retrospectively reviewed. The inclusion criteria were: (1) Clinically confirmed neurosyphilis, (2) All patients underwent MRI plain and enhanced examinations and/or functional examination.

Patients were diagnosed with neurosyphilis based on a combination of clinical symptoms, cerebrospinal fluid (CSF) analysis, and serological tests. The diagnostic criteria included: (1) A positive *T. pallidum* particle agglutination (TPPA) test in serum and/or CSF; (2) A positive CSF-Venereal Disease Research Laboratory (VDRL) test. (3) Elevated CSF white blood cell count (>5 cells/μL) or protein levels (>45 mg/dL) in conjunction with neurological symptoms.

### Image acquisition

All pretreatment MRI studies were performed using a 3-Tesla MRI scanner (Siemens Magnetom Trio, Germany; GE Discovery MR750, United States), using the same 8-channel head coil. The pretreatment MRI protocol included the following images: (a) Axial T1-weighted images (T1WI) (TR 1800 ms, TE 9.2 ms, FOV 240 × 240 mm, slice thickness 5 mm, intersection gap 1 mm, acquisition matrix = 256 × 256);(b) Axial T1-weighted images (T2WI) (TR4000 ms, TE 99 ms, FOV 240 × 240 mm, slice thickness 5 mm, intersection gap 1 mm, acquisition matrix = 256 × 256); (c) FLAIR imaging (TR 5500 ms, TE93 ms, FOV 240 × 240 mm, slice thickness 5 mm, intersection gap 1 mm, acquisition matrix = 256 × 256); (c) Axial DWI (TR 5000 ms, TE 73.2 ms (*b* = 1,000), FOV 220 × 220 mm, slice thickness 5 mm, intersection gap 1 mm, acquisition matrix = 128 × 128); (d) Contrast-enhanced T1-weighted imaging (T1WI-CE) (TR 6.3 ms, TE 3.1 ms, FOV 240 mm, slice thickness 1 mm, acquisition matrix = 192 × 192 matrix), performed after intravenous administration of gadolinium-based contrast agents.

### Image analysis and measurement

The MRI data were retrospectively analyzed by two senior radiologists. MRI images were examined for lesion morphology size, location, signal, borders, perilesional edema, and enhancement. Quantitative analysis included lesion volume measurement, signal intensity ratios (SIRs) on T2WI and FLAIR, and the degree of perilesional edema. For cases with contrast enhancement, the enhancement ratio (ER) was also calculated to evaluate vascular permeability changes.

Neurosyphilis is clinically divided into asymptomatic neurosyphilis, syphilitic meningitis, vascular syphilis, tabes dorsalis, and paralytic dementia. We classified neurosyphilis into four types according to clinical classification, extent of involvement, and MRI findings: parenchymal type, meningovascular type, syphilis gumma type, and Mixed type.

### Statistical analysis

Statistical analyses were performed using SPSS 25.0. Continuous variables were expressed as mean ± standard deviation (SD).

## Results

### Patient clinical characteristics

Of the 23 cases of neurosyphilis, including 19 males and 4 females, the mean age was 49.2 ± 11.4 years (range: 27 to 67). There were 16 cases of parenchymal type, mainly manifested as progressive cognitive impairment with psycho-behavioral abnormalities. Three cases of the meningovascular type presented with ischemic stroke with short duration and acute onset. Three cases of syphilis gumma type had a long course of disease and mainly presented with headache. Mixed type in 1 case, presented with headache (Table 1).

**Table 1:** Clinical characteristics of neurosyphilis patients.

Variables	Parenchymal type (*n* = 16)	Meningovascular type (*n* = 3)	Syphilis gumma type (*n* = 3)	Mixed type (*n* = 1)
Age range (years)	27–67			
Mean ages (years)	52 ± 8.9	46 ± 15.5	48.3 ± 13.1	28
Gender				
Men	12	3	3	1
Women	4	0	0	0
Clinical symptoms	Progressive cognitive impairment, psycho-behavioral abnormalities.	Ischemic stroke, short duration and acute onset	Headache, had a long course of disease	Headache

### Patient MRI characteristics

#### Parenchymal type

**Figure 1:**
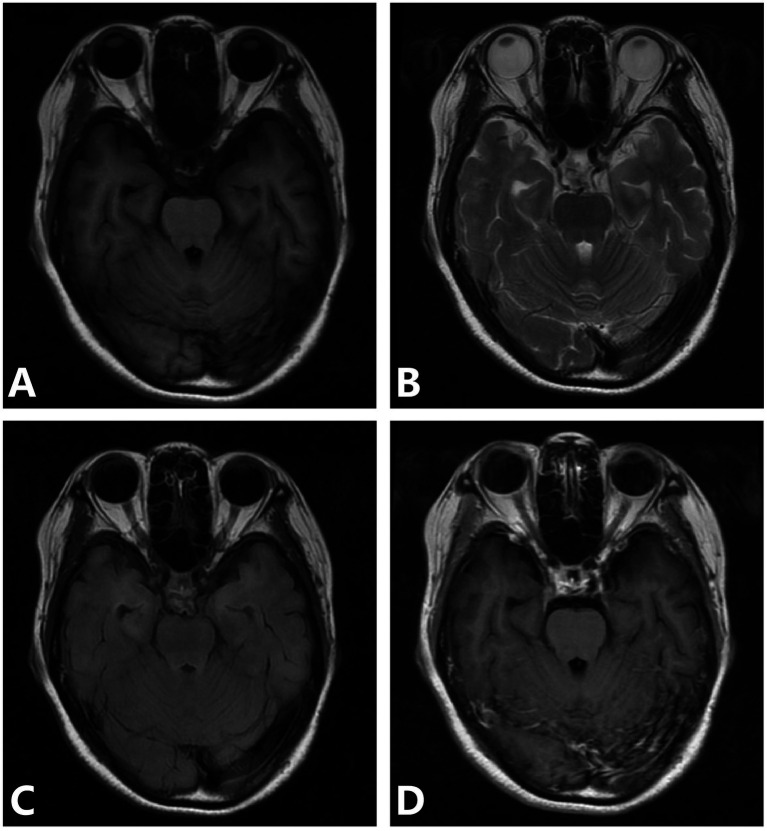
**(A–D)** Parenchymal type neurosyphilis, a 56-year-old female presented with sudden onset of unresponsiveness and difficulty in speaking for 1 day. MRI showed bilateral temporal lobe and hippocampal atrophy, increased signal intensity on T2WI and FLAIR, and no enhancement on enhanced scan. Panel **(A)** (T1WI): shows bilateral temporal lobe atrophy; panel **(B)** (T2WI): displays increased signal intensity in the hippocampal region; panel **(C)** (FLAIR): demonstrates hyperintense lesions with atrophy; panel **(D)** (T1WI-CE): shows no significant enhancement.

The lesions mainly showed bilateral temporal lobe and hippocampal atrophy, and the lesions could also involve the frontal lobe, parietal lobe, occipital lobe, and insula. The mean lesion volume was 4.8 ± 2.1 cm^3^, with a mean SIR of 1.52 ± 0.34 on FLAIR sequences. Perilesional edema was observed in 70% of cases, with an average edema-to-lesion ratio of 2.3 ± 0.8. T2WI and FLAIR showed hyperintense, DWI showed no diffusion restriction, and the lesions showed patchy enhancement or no enhancement on contrast-enhanced scans. All 10 cases had bilateral ventricular temporal horn dilatation and widening of the Sylvian fissure cistern (Figure 1).

#### Meningovascular type

**Figure 2:**
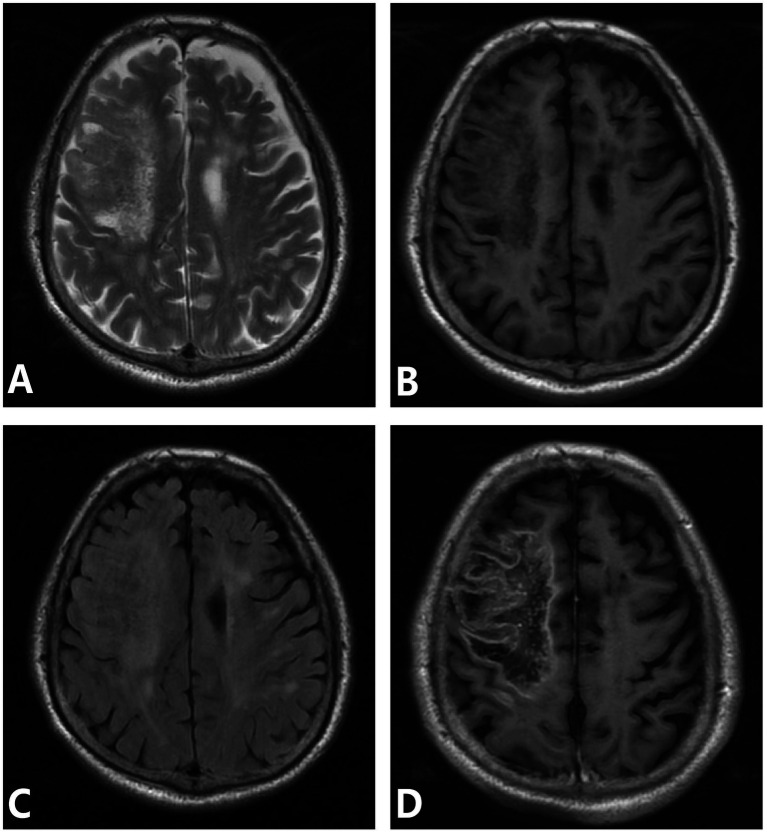
**(A–D)** Meningovascular type neurosyphilis, a 65-year-old male presented with episodic limb twitching for 1 month and recurrence with left limb weakness for more than half a month. MRI showed abnormal lesions in the right frontoparietal lobe, which showed low signal intensity on T1WI, heterogeneous high signal intensity on T2WI and FLAIR, and gyral and geographic enhancement on enhancement. Panel **(A)** (T2WI): shows heterogeneous high signal intensity; panel **(B)** (T1WI): shows low signal intensity in the right frontoparietal lobe; panel **(C)** (FLAIR): depicts hyperintense lesions with edema; panel **(D)** (T1WI-CE): demonstrates gyral and geographic enhancement.

One case mainly involved the brainstem, cerebellar hemisphere, and occipital lobe. MRI showed patchy and strip abnormal signals, hypointense on T1WI, hyperintense on T2WI and FLAIR, and restricted diffusion on DWI. One case involved the left basal ganglia, paraventricular, and left frontotemporoparietal cortex. MRI mainly showed patchy abnormal signals, hypointensity on T1WI, patchy hyperintense inside, hyperintense on T2WI and FLAIR, restricted DWI diffusion, patchy heterogeneous enhancement of lesions in the left basal ganglia and paraventricular region on enhancement, and gyral enhancement in the left frontotemporoparietal lobe. One case involved the right frontal lobe and showed low signal intensity on T1WI, high signal intensity on T2WI and FLAIR, heterogeneous high signal intensity on DWI, and geographic enhancement on enhancement (Figure 2).

#### Syphilis gumma type

**Figure 3:**
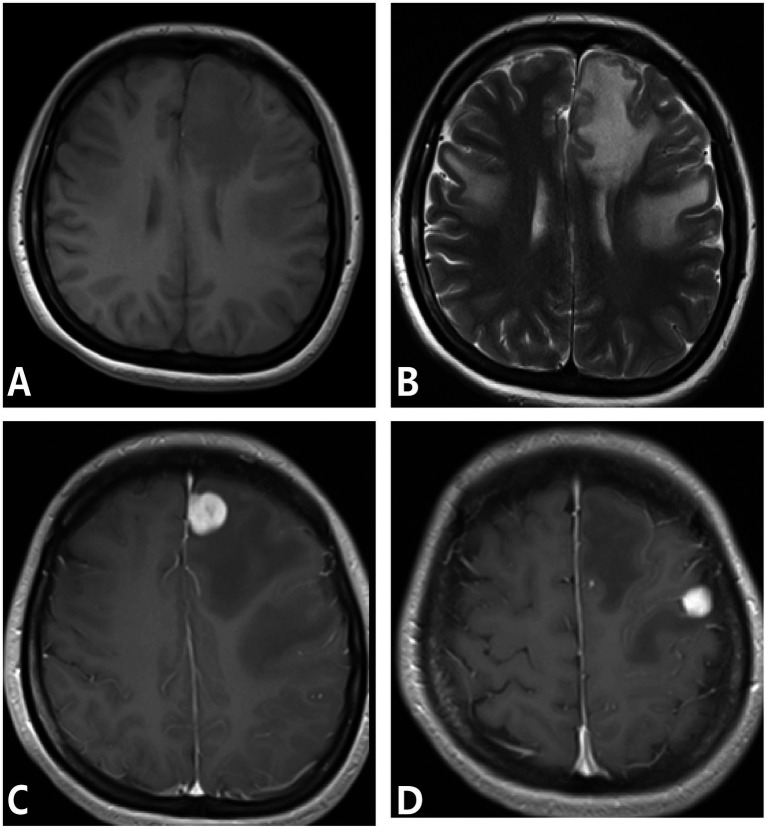
**(A–D)** Syphilis gumma type neurosyphilis, a 39-year-old male presented headache for 7 months, with paroxysmal right facial twitching for 1 month. MRI showed multiple lesions in the left frontal lobe surrounded by an edematous zone with marked enhancement. Panel **(A)** (T1WI): shows isointense mass-like lesions; panel **(B)** (T2WI): depicts hyperintense nodular lesions (arrow) with peripheral edema; Panel **(C)** (T1WI-CE): shows contrast-enhanced nodular lesions with surrounding edema; panel **(D)** (T1WI-CE): demonstrates marked nodular enhancement and adjacent meningeal enhancement.

**Figure 4:**
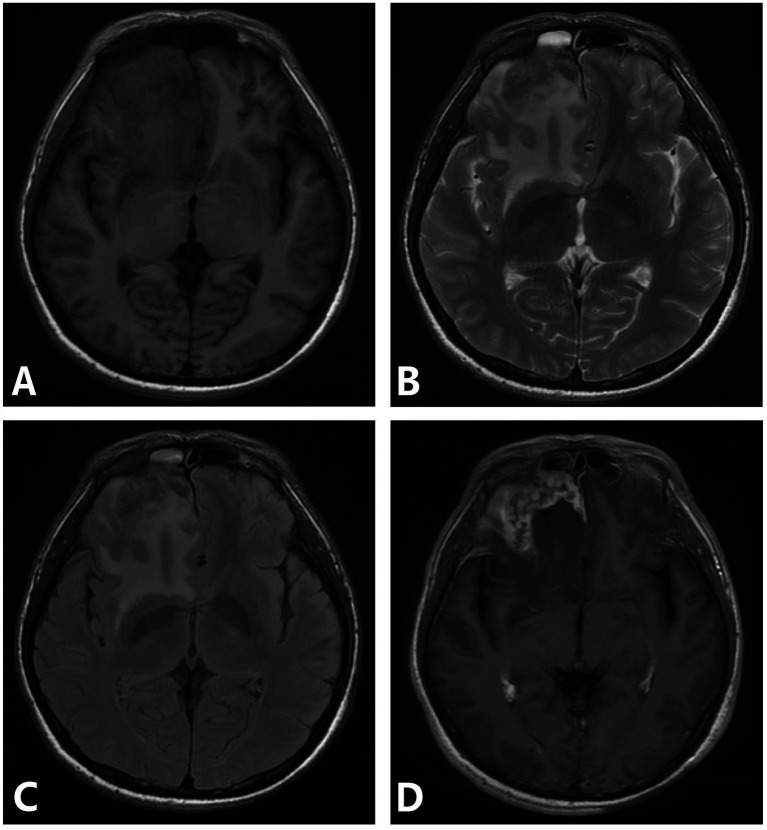
**(A–D)** Syphilis gumma type neurosyphilis, a 39-year-old male presented with intermittent suspiciousness and behavioral abnormalities for 4 days. MRI revealed an irregular lesion in the right frontal region with a surrounding edematous zone, showing heterogeneous signal intensity and marked heterogeneous enhancement with adjacent meningeal involvement. Panel **(A)** (T1WI): demonstrates an irregular isointense lesion in the right frontal lobe; panel **(B)** (T2WI): shows hyperintense signal within the lesion and surrounding edema; Panel **(C)** (FLAIR): depicts extensive hyperintense signals highlighting perilesional edema; Panel **(D)** (T1WI-CE): reveals marked heterogeneous enhancement with adjacent meningeal enhancement.

One case involved bilateral frontal and right temporal cortex and subcortex. MRI showed nodular and mass-like lesions, isointense on T1WI, slightly hyperintense on T2WI and FLAIR, and large patchy edema bands around them, and the enhanced lesions were significantly enhanced, adjacent meningeal enhancement. One patient had involvement of the left frontal and temporal lobes, right thalamus, and bilateral cerebral peduncles. MRI mainly showed nodular and mass-like lesions, isointense on T1WI, slightly hyperintense on T2WI and FLAIR, large patchy edema bands around them, and ring significant enhancement of enhanced lesions (Figure 3). One patient involved the right frontal lobe and adjacent meninges and showed irregular lesions, which showed low signal intensity on T1WI and heterogeneous high signal intensity on T2WI, with significant enhancement and adjacent meningeal enhancement (Figure 4).

#### Mixed type

One patient had involvement of the cerebellum, cerebral peduncle, periaqueductal region of the midbrain, thalamus, bilateral hippocampus, corpus callosum, and pineal gland. MRI mainly showed increased signal intensity on T2WI and FLAIR, and the pineal gland was significantly enhanced on contrast-enhanced scans, and no enhancement was observed in the other lesions. No obvious signs of atrophy were observed in the brain parenchyma.

## Discussion

This study systematically analyzed the clinical and MRI characteristics of 23 patients with neurosyphilis and highlighted several important findings. Among the four identified subtypes, the parenchymal type was the most common, accounting for nearly 70% of cases, and was predominantly associated with progressive cognitive decline and psychiatric abnormalities. MRI manifestations varied across subtypes: parenchymal neurosyphilis typically presented with bilateral temporal lobe and hippocampal atrophy accompanied by FLAIR hyperintensity; the meningovascular type was characterized by multiple acute infarctions with scattered distribution; syphilitic gummas appeared as mass-like lesions with marked perilesional edema and strong or ring enhancement; and the mixed type demonstrated overlapping and complex features. These results emphasize the heterogeneity of neurosyphilis and the potential value of MRI in improving diagnostic precision and guiding differential diagnosis.

In our cohort, the parenchymal type was the most frequent form of neurosyphilis (16/23, 69.6%), presenting predominantly with progressive cognitive decline and psychiatric abnormalities. MRI consistently demonstrated bilateral temporal lobe and hippocampal atrophy with FLAIR hyperintensity, sometimes extending to the frontal and parietal lobes. These findings align with previous studies describing temporal lobe involvement as the hallmark of parenchymal neurosyphilis (11, 12). The presumed mechanism involves *T. pallidum*–induced vasculitis and obliterative endarteritis, leading to neuronal loss and progressive atrophy (13, 14). Importantly, the gradual onset and bilateral atrophy in our patients contrast with viral encephalitis, which usually presents acutely with unilateral involvement, and autoimmune encephalitis, which shows bilateral hyperintensity without early atrophy and can be confirmed by antibody testing (13–15). Thus, our data support the role of MRI, in combination with serology, as a reliable diagnostic tool for parenchymal neurosyphilis (10, 16).

Tuberculous granulomas usually appear as thick-walled ring-enhancing lesions, often in the basal ganglia or brainstem, with frequent basal meningeal involvement. Cerebral toxoplasmosis, mainly in immunocompromised hosts, presents as multiple ring-enhancing lesions with edema at the gray–white junction, showing peripheral diffusion restriction (17). Cryptococcal infection typically produces gelatinous pseudocysts or nodular enhancement in the basal ganglia with minimal edema (18). By contrast, neurosyphilis often demonstrates bilateral temporal lobe atrophy, parenchymal or meningeal enhancement, and positive CSF serology, which together provide key diagnostic clues.

Three patients (13.0%) in our series were diagnosed with meningovascular neurosyphilis, all of whom presented with acute ischemic stroke. MRI revealed multiple infarcts with scattered distribution across different vascular territories, a pattern consistent with syphilitic arteritis rather than atherosclerosis. Previous reports indicate that up to 80% of patients initially presenting with stroke may later be identified as having neurosyphilis (19, 20), and our findings support this observation. The underlying pathophysiology involves *T. pallidum*–mediated vascular wall damage, collapse, and occlusion, resulting in obliterative arteritis or periarteritis (21–23). Clinically, this subtype is often misdiagnosed as atherosclerotic infarction; however, in contrast to our younger patients with diffuse lesions and positive serology, atherosclerotic infarction is typically restricted to arterial territories in older, seronegative individuals. These findings emphasize the need to include neurosyphilis in the differential diagnosis of atypical or multifocal strokes.

Syphilitic gumma accounted for three cases (13.0%) in our cohort, all with a long disease course and predominant headache symptoms. MRI demonstrated mass-like or nodular lesions with marked perilesional edema, strong or ring enhancement, and adjacent meningeal thickening. These results are consistent with earlier reports describing gummas as tumor-like lesions associated with granulomatous inflammation and caseous necrosis (24). However, our cases highlight the diagnostic challenge of differentiating gummas from intracranial tumors or infectious granulomas. For instance, brain metastases are usually located at the gray–white matter junction with a history of primary cancer, while Rosai–Dorfman disease (RDD) shows a long, thick meningeal tail sign (25, 26). In contrast, syphilitic gummas in our patients were located in the convexity and demonstrated shorter tails with prominent edema. Similarly, tuberculous granulomas and cryptococcal lesions typically involve the basal ganglia and show different enhancement patterns (18, 19). Cerebral toxoplasmosis, mainly seen in immunocompromised hosts, may present with the eccentric target sign, which was absent in our cases (27). These distinctions underline the importance of integrating MRI patterns with clinical context and serological findings to avoid misdiagnosis.

However, this study has certain limitations, particularly the relatively small sample size (23 cases), which may limit the generalizability and reliability of the identified imaging patterns. Given the rarity of neurosyphilis and the challenges associated with recruitment, this study serves as a preliminary exploration rather than a definitive conclusion. Further multi-center studies with larger patient cohorts are necessary to validate our findings and assess their diagnostic value.

## Conclusion

In conclusion, the clinical and MRI manifestations of neurosyphilis are diverse. Quantitative analysis demonstrated that lesion volume, perilesional edema, and signal intensity ratios could aid in differentiating subtypes. The parenchymal type was associated with larger lesion volumes and higher SIRs on FLAIR, while the meningovascular type exhibited greater diffusion restriction. These findings suggest that incorporating quantitative imaging biomarkers may improve diagnostic accuracy and facilitate early intervention. Given the absence of pathological confirmation in most cases, future prospective studies should aim to include histopathological data or consistent clinical follow-up to enhance the reliability of the identified imaging features.

## Data Availability

The original contributions presented in the study are included in the article/supplementary material, further inquiries can be directed to the corresponding authors.
